# Investigating the Barriers Faced by Biomedical Science Undergraduates in Completing a Placement Year

**DOI:** 10.3389/bjbs.2026.14947

**Published:** 2026-02-05

**Authors:** Kathryn Dudley, Amreen Bashir

**Affiliations:** 1 School of Pharmacy and Life Sciences, Faculty of Science and Engineering, University of Wolverhampton, Wolverhampton, United Kingdom; 2 School of Biosciences, College of Health and Life Sciences, Aston University, Birmingham, United Kingdom

**Keywords:** barriers, biomedical science, employability, IBMS registration portfolio, placement

## Abstract

**Introduction:**

Research shows completing a placement year is associated with improved academic and employment outcomes. For Biomedical science courses, pathology placements allow completion of the Institute of Biomedical Science (IBMS) registration training portfolio and obtaining Health and Care Professions Council (HCPC) registration post-graduation. This study sought to identify the barriers biomedical science students across the West Midlands region of England face when completing a placement year, to identify strategies which promote inclusivity to overcome these barriers.

**Materials and Methods:**

Level 5 and Level 6 students from Aston, Coventry, Keele and Wolverhampton universities were invited to complete a questionnaire which included a mixture of Likert scale and free-text responses. A range of questions assessed student perceptions on the importance of placement opportunities, as well as identifying factors which were important when pursuing a placement year. Likert scale data was analysed quantitatively, and a Mann Whitney U or Kruskal Wallis test were used to infer significance, whereas free text responses were analysed using thematic analysis.

**Results:**

A total of 107 students completed the questionnaire. Students who declared a disability were less likely to undertake an unpaid placement compared to their peers (p = 0.013). Of those students who declared caring responsibilities, 33.3% chose not to apply for a placement year compared to 18.2% of those who did not have caring responsibilities (p = 0.020). Participants reported that funding was important when deciding whether to pursue a placement (88.8%). Thematic analysis revealed several recurring themes deterring student placement applications, including financial support and placement availability within their geographical area. Students valued the importance of professional recognition following the placement and the development of technical and transferable skills.

**Discussion:**

Many of the barriers are fuelled by financial constraints which deter students from applying to placement positions. Despite the need to increase the Biomedical Scientist workforce, the strategies to increase training opportunities are not well established. Equity in placement funding from centralised sources is key to ensuring Biomedical Scientists can excel in their professional careers. Through availability of funding, marginalised populations will have the same opportunities as their peers therefore producing more employable graduates to meet pathology workforce demands.

## Introduction

A Biomedical Science Bachelors (BSc) is a highly sought after degree programme in the United Kingdom (UK) offering an excellent foundation for numerous post-graduate career opportunities. Many graduates choose to work within the National Health Service (NHS) as a Biomedical Scientist upon obtaining registration with the Health and Care Professions Council (HCPC). Other post-graduate opportunities exist within the NHS, including roles such as a Physician Associate (PA), Scientist Training Programme (STP), clinical researchers and medical laboratory assistants (MLA). Additionally, graduates may explore opportunities beyond the healthcare sector including careers in industrial research, education or through further study (including post-graduate medicine or Masters degree courses).

Biomedical Science courses that are accredited by the Institute of Biomedical Science (IBMS) include optional placement opportunities for all BSc Biomedical Science students. In some courses, mandatory placements are integrated throughout the three-year degree course as part of the Practitioner Training Programme (PTP) [1] or an apprenticeship, whilst in other courses these placements lie at the end of the second year of the undergraduate degree programme. Students can choose between an NHS laboratory placement within an IBMS approved training laboratory [2] (typically unpaid) and an industrial sandwich placement (typically paid). Students must decide whether a placement opportunity aligns with their career aspirations and choose the most suitable pathway based on their post-graduate goals. However, due to limited availability, placement opportunities are extremely competitive.

The West Midlands Applied Biomedical Science programme has been developed in England to meet the workforce needs of regional NHS pathology laboratories. This collaborative approach involves the integration of a placement at the end of the second year of study in an IBMS approved training laboratory. Annually, around 40 regional students obtain laboratory-based training and complete the IBMS Registration Training portfolio allowing students post-graduation to register with the HCPC. Currently, five Higher Education Institutions (HEIs) including Aston, Coventry, Keele, Staffordshire and Wolverhampton universities have established a strong partnership with Training Officers (TOs) from local Trusts, encompassing over twenty hospitals. This partnership includes combined placement workshops accessible to all students undertaking the Applied Biomedical Science programmes. This coordinated provision of placements benefits all stakeholders. Typically, NHS laboratory placements are full-time unpaid positions, with students being given a study day ‘off the bench’. Although placement opportunities at the end of the second year of the award are open to all students, placement availability is limited.

To pursue a career as a Biomedical Scientist, candidates must complete both an IBMS accredited degree course [3] and a period of in-house laboratory training in an IBMS approved training laboratory, leading to successful award of the IBMS Certificate of Competence. Candidates who lack one or both requirements will not be eligible to obtain HCPC registration as a Biomedical Scientist. Following the completion of this period of laboratory training and the IBMS Registration Training portfolio, candidates are assessed by an independent IBMS verifier [4] to ensure that they meet the necessary HCPC Standards [5, 6] to practice as a Biomedical Scientist. For graduates seeking a career as a Biomedical Scientist, NHS laboratory placements or degree apprenticeships offer the opportunity to complete the IBMS Registration Training portfolio before graduating, which provides a significant advantage when seeking employment. Candidates who do not complete the IBMS Registration Training portfolio during their degree must apply for trainee Biomedical Scientist positions, or increasingly MLA positions whilst awaiting the opportunity to complete the IBMS Registration Training portfolio. This pathway to achieving HCPC registration is prolonged due to the limited availability of trainee Biomedical Scientist positions.

### Benefits of Completing a Sandwich Placement

The benefits of placements for future employability are well recognised due to the development of transferable skills, better performance in final year studies and the ability to obtain graduate employment [7–9]. One study demonstrated that following completion of a placement year, students gain an average of almost 4% in their final year grade point average [10]. However, there is some debate about whether this increase in performance is achieved because high-calibre candidates tend to apply for and successfully complete a placement year and were therefore destined to be high achievers [11]. Within the West Midlands, previous graduates of the Applied Biomedical Science programmes often return to their placement provider as a paid employee post-graduation. This is something which is common across a range of placement opportunities, as this year allows employers to fully assess an individual’s capabilities to fulfil their role [12, 13].

For graduates of BSc Biomedical Science, the completion of the IBMS Registration Training portfolio allows graduates to join the HCPC register as a Biomedical Scientist, which is a mandatory requirement for employment in this role within the NHS and private healthcare organisations. Often, laboratory experience is required when applying for graduate roles and a lack of experience in a professional laboratory makes obtaining graduate employment challenging [14]. Completion of a placement year involves significant benchwork where students develop their practical laboratory skills [10, 15]. Undergraduate placements are important for providing a link between theory and practice, and this application of theory to a real-world setting often leads to improved academic achievement [7].

Highly skilled graduate employability figures are significantly higher for those students who complete a 4-year Biomedical Science course, which consists of 3 years of study and an integrated 12-month placement. Data shows that completion of the IBMS Registration Training portfolio and registration with the HCPC leads to highly skilled graduate employability rates of almost 100% [16]. Graduates of science degree courses should be prepared for life-long learning, not only learning the threshold concepts needed to fulfil their role but also mastering their subject area and keeping their knowledge and skills up to date [17]. This requirement is also essential for Biomedical Scientists who must participate in Continuing Professional Development (CPD), as this is a requirement for all HCPC registered professionals [18]. Placement years provide the opportunity to engage in CPD activities within the workplace and reinforce the importance of these for future professional development.

### Challenges for Students in Pursuing Placement Opportunities

Despite the numerous and well evidenced benefits of completing a placement year, anecdotal evidence suggests that some students wish to apply but are unable to do so due to financial pressures, caring responsibilities, associated travel costs, travel duration and challenges associated with full time work. In the West Midlands area, Applied Biomedical Science placements are available only to students who study full time due to the requirement to develop the knowledge and skills to practice as a Biomedical Scientist. It is well recognised that this is a significant undertaking, with the typical duration for completing the Registration Training portfolio being upwards of 6 months, and typically around 1 year. Despite a reduction in university tuition fees for placement year, some students report a lack of feasibility according to their individual circumstances. In the current financial climate, many students are concerned about accruing debt during their undergraduate studies, and for some students extending their studies by an additional year to complete a placement is financially challenging or even impossible [7]. Since the removal of the Higher Education Funding Council for England (HEFCE) bursary for the NHS Applied Biomedical Science placement year in 2012, some universities have reported a reduction in the number of students who choose to pursue a placement [16].

For those students wishing to complete a placement in an IBMS approved training laboratory and complete their IBMS Registration Training portfolio, placement availability is a significant challenge [19]. Typically, only around 10% of students on IBMS accredited Biomedical Science courses will obtain a placement [16]. Often students apply to Trusts which are further from their home address to increase their chances. However, students are often unable to afford accommodation near to placement [16], requiring a lengthy and costly commute and extending their working day further. This provides additional financial pressure and risks students becoming burnt out. In addition, the support received from academic staff in advising students on placement opportunities is known to influence decisions to pursue a placement. Poor advice and a lack of support from academic staff are known to be major factors in students choosing not to pursue a biosciences placement [20]. Therefore, experienced and knowledgeable academic staff are instrumental to supporting students interested in completing a placement year.

### Aims and Objectives

This study aimed to identify the factors which influence students’ decisions to pursue a placement year and barriers to obtaining placements during their IBMS accredited Biomedical Science degree programmes within the West Midlands area. Although placement applications for the Applied Biomedical Science course are highly competitive, anecdotal evidence suggests that some students choose not to pursue this route, despite their career aspiration being to become a Biomedical Scientist. By identifying the factors which may prevent students from engaging with placement opportunities, the study seeks to make recommendations for changes to practice and policy to overcome these barriers.

## Materials and Methods

Students from Aston, Coventry, Keele and Wolverhampton universities participated in this study. Students were invited to complete a questionnaire, which included a mixture of Likert scale and free-text responses. Two distinct questionnaires were developed to gather insights on student decision-making regarding placement years. The first questionnaire targeted Level 5 (second year) students deciding whether to undertake a placement year. The second questionnaire was designed for Level 6 (third year) students to explore the factors influencing their decision to pursue or forgo a placement. All students were eligible to participate, regardless of whether they had completed a paid industrial placement, an unpaid NHS laboratory placement, been unsuccessful in securing a placement, or chosen to progress directly to their final year.

Students were asked a range of questions to gauge their perceptions of placement opportunities, as well as identify the factors which were important when deciding whether to pursue a placement year. Students scored their agreement with several statements using a 5-point Likert scale (Strongly agree, agree, neither agree nor disagree, disagree and strongly disagree) and had the opportunity to complete several free-text responses. To facilitate participation, a QR code and weblink to the questionnaire were shared with students at both levels via the virtual learning environment (VLE) at each of the four participating universities. Where possible, students were given time during a taught session to allow them to participate in the study. The questionnaire launched in February 2025 and was open for 4 weeks. Students were sent a final participation reminder 7 days before the questionnaire closed.

### Ethical Approval

The questionnaire was administered via JISC Online Surveys and the participant information sheet and informed consent form were embedded. Participants were made aware in the participant information sheet that they could omit any questions they did not wish to answer. The only mandatory questions were those within the consent form. Ethical approval was obtained from Aston University School of Health and Life Sciences Ethics Committee (REC ID: HLS21247), Coventry University Faculty of Health and Life Sciences Research Ethics Committee (REC ID: P185310), Keele University Research Ethics Committee (REC ID: 1014) and University of Wolverhampton Life Sciences Ethics Committee (REC ID: LSEC/2024-25/KD/32). Participants did not provide any identifiable information as part of the study and were informed that once they submitted their responses, their anonymised data could not be removed from the study.

### Data Analysis

Data was analysed using statistical analysis of the quantitative data (e.g., frequency data, mean and standard deviation as appropriate). Likert scale data was converted to a numerical scale to facilitate statistical analysis via non-parametric methods (1 = strongly agree, 2 = agree, 3 = neither agree nor disagree, 4 = disagree, 5 = strongly disagree). To compare the level 5 and level 6 student responses, Mann Whitney U and Kruskal Wallis tests were carried out using IBM-SPSS Statistics version 29. Statistical significance was set as *p* < 0.05. Qualitative data was analysed using thematic analysis according to Braun and Clarke to identify significant themes [21]. The researchers read the data for familiarity, generated codes to form initial themes, and checked for plausibility. The process was repeated by both researchers, and the final themes were collectively agreed upon to produce the thematic analysis.

## Results

### Student Demographics

A total of 107 students enrolled in Biomedical Science courses across four higher education institutions; Aston University, Coventry University, the University of Wolverhampton, and Keele University completed the questionnaire. The cohort included 63 Level 5 and 44 Level 6 students. The majority of participants reported their ethnic origin as the Indian Sub-continent (40.2%) or White European (26.1%). The response rate for the study was moderate, with 384 students at Level 5 and 281 students at Level 6 invited to participate. This represents a response rate of 16.4% at Level 5% and 15.7% at Level 6, which is discussed further in the limitations of the study. Although the participants represented four different HEIs, the student demographics across the participants were broadly similar and a breakdown of participants by key demographic groups is shown in Table 1.

**TABLE 1:** An overview of the demographic data for the participants in the study, grouped according to level of study.

Category	Sub-group	L5 students		L6 students	
		Number	%	Number	%
Host institution	Aston University	15	23.8%	17	38.6%
	Coventry University	2	3.2%	6	13.6%
	Keele University	19	30.2%	5	11.4%
	University of Wolverhampton	27	42.9%	16	36.4%
Caring responsibilities	Declared	5	7.9%	8	18.2%
	Not-declared	58	92.1%	36	81.8%
Disability or learning difference	Declared	8	12.7%	7	15.9%
	Not-declared	55	87.3%	37	84.1%
Ethnicity	Middle Eastern	3	4.8%	0	0.0%
	Indian Sub-Continent	24	38.1%	19	43.2%
	White European	14	22.2%	14	31.8%
	African/African Caribbean	8	12.7%	5	11.4%
	South East Asia and China	6	9.5%	1	2.3%
	Other	3	4.8%	1	2.3%
	Prefer not to say	5	7.9%	4	9.1%

Of the Level 5 students, 34.9% wished to pursue an unpaid NHS placement, 14.3% wished to pursue a paid sandwich placement and 15.9% could not decide which type of placement to apply for. A further 27.0% of Level 5 students did not apply to complete a placement, whilst 6.4% of respondents applied but were unsuccessful. One Level 5 participant omitted their response to this question (1.6%). Overall, for Level 5 participants, 65.1% aspired to complete a placement year. At the time the questionnaire was distributed, Level 5 students had not yet completed the NHS placement selection process.

Of the Level 6 participants, 31.8% had completed an unpaid NHS placement year, whilst 15.9% had completed a paid industrial placement. A further 9.1% had applied for a placement year but been unsuccessful. The Level 6 participants included 31.8% who had chosen not to apply for a placement year and a further 3 participants (6.8%) omitted their response to this question. Finally, 2 (4.5%) Level 6 students provided free text responses to explain their placement status, including one student who wanted to apply but could not due to caring responsibilities and one student who applied and obtained a placement but could not complete the placement due to financial pressures. Across both cohorts, 49.1% of participants wished to or had completed a placement year.

### Important Factors for Students Deciding Whether to Complete a Placement Year

**FIGURE 1:**
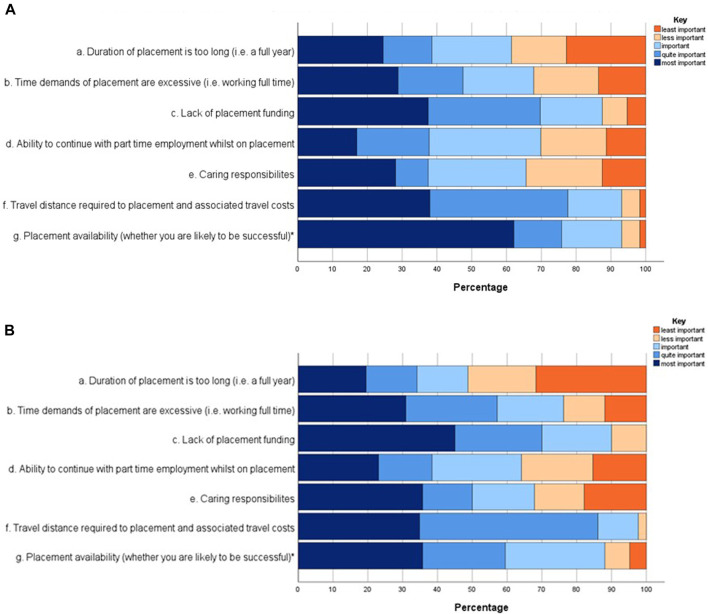
**(A)** Level 5 (n = 63) student responses rating the importance of individual factors when applying for a placement. A five-point Likert scale was used to answer each statement, where 1 is most important and 5 is least important. A Mann-Whitney U test was used to determine statistical significance (*p < 0.05) between the Level 5 and 6 student cohorts. **(B)** Level 6 (n = 44) student responses rating the importance of individual factors when applying for a placement. A five-point Likert scale was used to answer each statement, where 1 is most important and 5 is least important. A Mann-Whitney U test was used to determine statistical significance (*p < 0.05) between the Level 5 and 6 student cohorts.

Students were presented with a series of statements to score their agreement using a five-point Likert scale. Students were asked to rate seven statements from most important to least important, with the option to select not applicable. The student responses to these statements have been presented in Figures 1A,B. Students allocated each statement a number, with 1 being most important and 5 being least important.

In response to statement a, which considered the placement duration, 61.4% of Level 5% and 48.7% of Level 6 participants reported this was important when choosing to apply for a placement (*p* = 0.306). For statement b, which considered the full-time nature of the placement year, 67.7% of Level 5% and 76.2% of Level 6 students considered this to be an important factor when deciding whether to complete a placement (*p* = 0.451). A key finding was statement c, that 87.5% of Level 5% and 90% of Level 6 participants felt that lack of placement funding was important when deciding whether to pursue a placement (*p* = 0.557). Students also considered the ability to continue with part time work in statement d, where 69.9% of Level 5 and 64.1% of Level 6 students rated this as important in their decision making (*p* = 0.971). In response to statement e which considered the significance of caring responsibilities, 65.6% of Level 5 and 67.9% of Level 6 students considered these to be important (*p* = 0.642). For statement f, which considered travel distance to placement and the cost implications of this, a significant proportion of respondents felt this was key, with 93.1% of Level 5% and 97.7% of Level 6 students rating this as important (*p* = 0.756). In response to statement g which considered placement availability and whether students were likely to be successful, 93.1% of Level 5 students and 88.1% of Level 6 students felt this was important (*p* = 0.015). A higher proportion of Level 5 students (62.1%) felt that this was the most important factor when considering whether to apply for a placement year, achieving statistical significance between the two cohorts. It is evident that placement funding and the travel distance to placement, along with the associated funding implications of this, are the most important considerations for students.

### Assessing Students’ General Perceptions of a Placement Year

**FIGURE 2:**
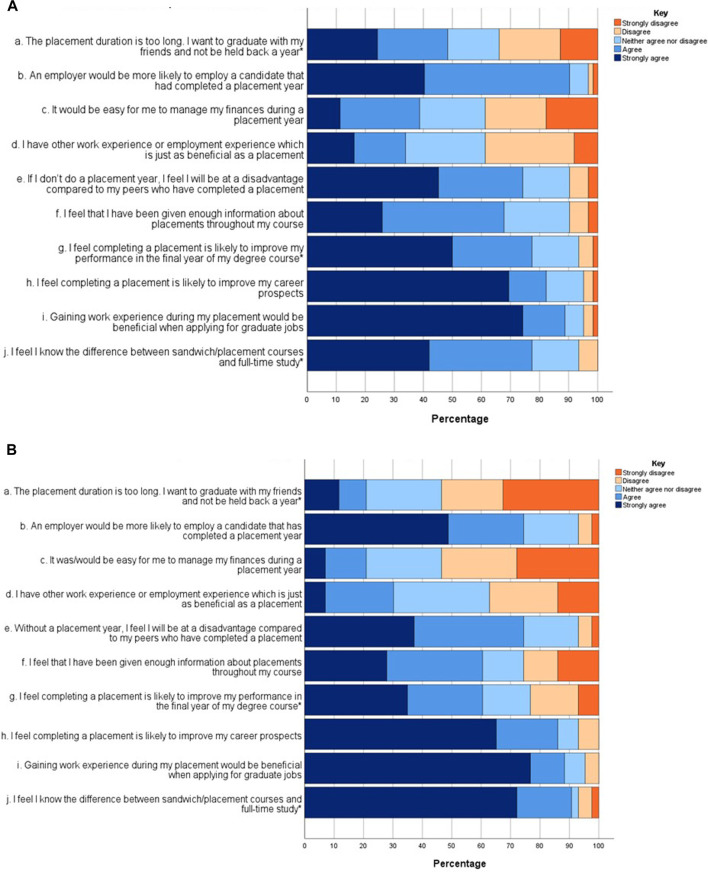
**(A)** Level 5 (n = 63) student responses rating agreement with a series of attitude statements. A five-point Likert scale was used to answer each statement, where 1 is strongly agree and 5 is strongly disagree. A Mann-Whitney U test was used to determine statistical significance (*p < 0.05) between the Level 5 and 6 student cohorts. **(B)** Level 6 (n = 44) student responses rating agreement with a series of attitude statements. A five-point Likert scale was used to answer each statement, where 1 is strongly agree and 5 is strongly disagree. A Mann-Whitney U test was used to determine statistical significance (*p < 0.05) between the Level 5 and 6 student cohorts.

Students were asked to rate several statements relating to placements and employability from strongly agree to strongly disagree, with the option to also select a neutral response. The responses are presented in Figures 2A,B.

In response to statement a, which considered the placement duration, 48.4% of Level 5 but only 20.9% of Level 6 students agreed or strongly agreed that a placement year was too long (*p* = 0.005). This demonstrates a significant difference between the two cohorts when considering if a 12-month placement is an appropriate timeframe. For statement b, 90.3% of Level 5% and 74.4% of Level 6 students agreed that employers were more likely to employ a candidate that had completed a placement year (*p* = 0.879). Statement c asked whether students felt it would be easy to manage their finances during a placement year and 38.7% of Level 5% and 21.0% of Level 6 students agreed with this statement (*p* = 0.063). This suggests that all students have concerns regarding managing finances during placement year, however, Level 6 students were more aware of the financial challenges. For statement d, students were asked whether other work experience or employment was as beneficial as a placement, with only 33.8% of Level 5% and 30.3% of Level 6 students agreeing with this statement (*p* = 0.582). When asked whether they felt without a placement year they would be at a disadvantage (statement e), 74.2% of Level 5% and 74.2% of Level 6 students agreed with this statement (*p* = 0.638).

For statement f, students were asked whether they had been given enough information about placements during their course and 67.7% of Level 5% and 60.5% of Level 6 students agreed with this statement (*p* = 0.418). Statement g discussed whether final year degree performance was improved by completing a placement year and 77.4% of Level 5% and 60.5% of Level 6 students agreed with this statement (*p* = 0.035). This represents a statistically significant difference between the two cohorts. Statement h considered whether completing a placement improved career prospects and 82.3% of Level 5% and 86.0% of Level 6 students agreed with this statement (*p* = 0.771). In response to statement i, gaining work experience during placement would be beneficial when applying for graduate jobs, 88.7% of Level 5% and 88.3% of Level 6 students agreed with this statement (*p* = 0.793). For statement j, students determined whether they knew the difference between sandwich courses and full-time study, with 77.4% of Level 5% and 90.7% of Level 6 students agreeing with this statement (*p* = 0.004). This demonstrated a statistically significant difference between the two cohorts.

### Equality, Diversity and Inclusivity (EDI)

To consider the EDI implications of placement opportunities, further statistical analysis was carried out using the Mann Whitney U test and the Kruskal Wallis test to consider the impact of gender, ethnicity, disability and caring responsibilities on the likelihood of completing a placement year. For this analysis, the Level 5 and Level 6 responses were grouped to identify patterns across the entire cohort.

### Gender

There were 99 participants who declared their gender across both student cohorts, of which 24.2% identified as male and 75.8% identified as female. There were no participants who identified as non-binary or transgender. Of the male participants, 70.8% applied for a placement year, compared to 58.7% of female participants demonstrating a non-significant difference with the Mann Whitney U test (*p* = 0.266).

### Ethnicity

There were 98 participants who declared their ethnicity across the Level 5 and Level 6 participants. Of the participants, 13 (12.1%) were African or African Caribbean, 43 (40.2%) were from the Indian sub-continent, 3 (2.8%) were Middle Eastern, 7 (6.5%) were from Southeast Asia or China and 28 (26.2%) were White European. There were 4 (3.7%) participants who identified their ethnic origin as ‘other’ and these individuals were of mixed heritage. A further 9 participants (8.4%) opted for the ‘prefer not to say’ option. A Kruskal Wallis non-parametric test was performed to determine whether there was a difference between placement status according to ethnicity. Of those participants who were of African origin, 80% of participants applied for a placement. Amongst participants from the Indian sub-continent 72.5% applied for a placement year, compared to 66.7% of participants of Middle Eastern origin. Of the Southeast Asian or Chinese participants, 87.5% of participants applied for a placement. Amongst the White European students, 74.1% applied to complete a placement year. In the students who identified their ethnic origin as ‘other’, 80% of participants applied for a placement year. Of those participants who identified their ethnic origin as ‘prefer not to say’, 100% of participants applied for a placement year. The Kruskal Wallis test identified a non-significant difference between participant ethnicity and placement status (*p* = 0.320).

### Disability or Learning Difference

There were 103 responses received for the statement “if the placement year were paid, would this have influenced your decision to apply.” Data analysis revealed that 15 students (14.6%) declared a disability or learning difference. Of those students who declared a disability, 33.3% would apply whether placement was paid or unpaid. Of those students who did not declare a disability, 42.0% said they would apply whether the placement was paid or unpaid (*p* = 0.013). This represents a statistically significant difference using the Mann Whitney U test with fewer students who declare a disability choosing to apply for an unpaid placement. There were 101 responses received when considering the placement status of the participants (whether they had completed or wished to complete an NHS placement, industrial placement, were not selected or chose not to apply). These responses were analysed according to self-declared disability status. Of those students who declared a disability, 40.0% chose not to apply to complete a placement year, compared to 19.8% of students who did not declare a disability (*p* = 0.108). This represents a statistically significant difference based on self-declared disability status using the Mann Whitney U test.

### Caring Responsibilities

When asked about caring responsibilities, 100 responses were received, of which 13% of students declared caring responsibilities. Participants reported caring responsibilities for children, siblings, parents and grandparents. Of those students with caring responsibilities 58.3% declared they would apply for a placement if it were paid, but not unpaid. For the remaining students without caring responsibilities, 47.7% declared they would apply for a placement if it were paid, but not unpaid (*p* = 0.899). A total of 33.3% of students who declared caring responsibilities chose not to apply for a placement year compared to 18.2% of those who did not have caring responsibilities (*p* = 0.020), representing a statistically significant difference using the Mann Whitney U test. This suggests that those students who have caring responsibilities are less likely to apply for a placement year.

### Thematic Analysis of Free-Text Responses

**FIGURE 3:**
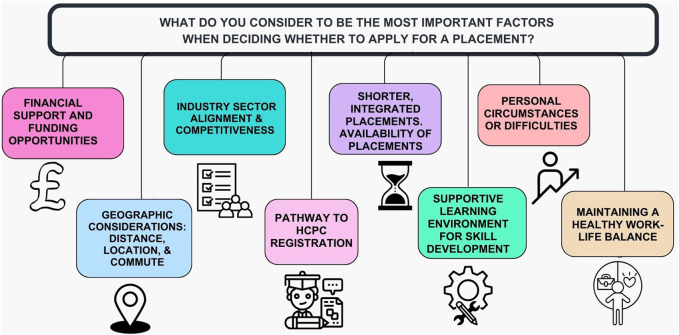
The most important factors for Level 5 and Level 6 students when deciding whether to apply for a placement.

**FIGURE 4:**
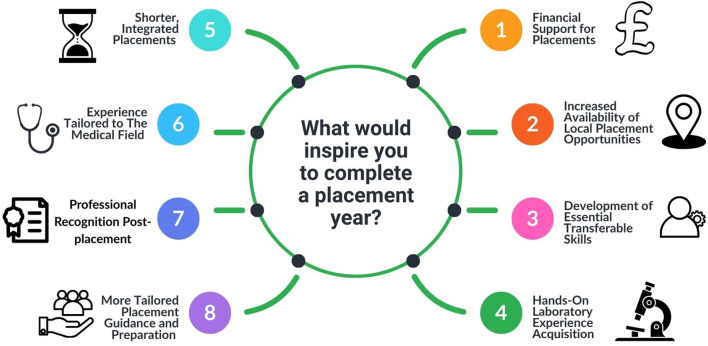
Key factors influencing Biomedical Science students’ motivation to complete clinical placements.

As part of the questionnaire, students were invited to provide expanded responses on the following topics: (i) the most important factors influencing their decision when applying for a placement (Figure 3), (ii) what would motivate them to undertake a placement year (Figure 4), and (iii) whether they recognised the benefits of completing a placement year. Thematic analysis of the responses revealed several recurring themes and several students’ responses contained more than one theme, with the four most prominent being:

- Access to financial support
- Availability of placements within their local geographical area
- Professional recognition following the placement
- Development of both technical and transferable skills

Respondents expressed strong opinions about the key factors that would motivate them to consider a placement opportunity (Figure 4). The free-text responses provided valuable insights, with students offering specific and detailed comments:

### Geographical Location and Flexibility

For many students, especially those with personal or family responsibilities, the practicality of a placement, its location and scheduling was a primary concern.

“Location - Has to be West Midlands based as I have children. Hours - How many hours are required. Paid/unpaid - I am open to an unpaid placement, however I still need to work a paid role part time.”

### Financial Constraints

A recurring theme in the responses was the financial burden associated with unpaid placements (Figures 3, 4). Many students highlighted how the lack of income made participation unfeasible, with some calling attention to the inequity this creates. Some respondents expressed frustration over the lack of financial support through NHS bursaries for Biomedical Science students.

“If I was paid decent money, I find it ridiculous how most full-time placements are unpaid. It discriminates between students who are lucky enough to come from a background with money vs those of us wanting opportunities but not applying because we cannot afford it. I have to cover travel expenses but also have an income that’s going to support me.”

“The type of placement I can apply to and whether this will give me opportunity to work in the NHS. If I would be paid as I would have to have quit my job and I would not be able to live for a year with no income.”

“NHS Bursary availability for financial support. (As someone who completed an NHS unpaid placement) I think it's actually disgusting to not include Biomedical students who secure NHS placement entitlement to a bursary.”

### Professional Registration and Accreditation

The prospect of earning qualifications or credentials that would aid in future employability was a powerful motivator. For many, placements served as a pathway toward completing essential requirements such as the IBMS Certificate of Competence and HCPC registration post-graduation.

“1. What I will gain at the end of the placement year (in this case, IBMS Certificate of Competence which will allow me to apply for HCPC registration after completing my degree). 2. If you will be able to financially survive. Since the NHS placements are unpaid, you need to consider whether you’ll be able to relocate to the hospitals you are applying to and if you are eligible for student finance to help you cover the costs of living during your placement”

“More prospects once I graduate and being HCPC registered.”

“You can graduate and work as Band 5 BMS—you do not have to start any lower, you can complete registration portfolio, exposure to real lab. (Getting work experience in a lab is not easy).”

### Post-Graduation Employment and Real-World Exposure

Many respondents viewed a placement as a critical step toward career development. These experiences were seen as invaluable opportunities to enhance skills, gain exposure to real-world laboratory environments, and make meaningful professional connections. There was a strong emphasis on the importance of placements for providing an opportunity to work within the NHS and pathology. The ability to apply theory in practice, understand the healthcare system, and working in multidisciplinary teams was seen as highly beneficial.

“Completing a placement year would inspire me because it would provide me with hands-on experience, aid in my career progression and also enhance my employability and boost my chances upon graduation in an increasingly competitive NHS job market.”

“It would allow me to complete my IBMS registration portfolio before having finished my university studies, gain invaluable practical skills and build professional connections that can lead to potential job opportunities in the future.”

“Completing a laboratory NHS hospital placement benefits are: on-hand laboratory experience, to understand the practical NHS system and how to apply healthcare protocols, quality controls in the lab and complying with the healthcare regulations, to enhance and develop technical skills and soft skills, to focus and contribute to patient care in practise and not only the theoretical way, and to increase confidence in the lab.”

“It would allow me to gain insight into NHS procedures, multidisciplinary work and the fast-paced nature within hospital labs, preparing me for the realities of the job. The placement would allow me to build professional connections with experienced Biomedical Scientists, increasing my chances of securing a permanent role upon graduation. A placement within the NHS overall gives me a strong foundation within the field, improving both my employability and confidence.”

“Developing professional skills & networking opportunities. Gaining hands-on experience in my field of interest.”

**FIGURE 5:**
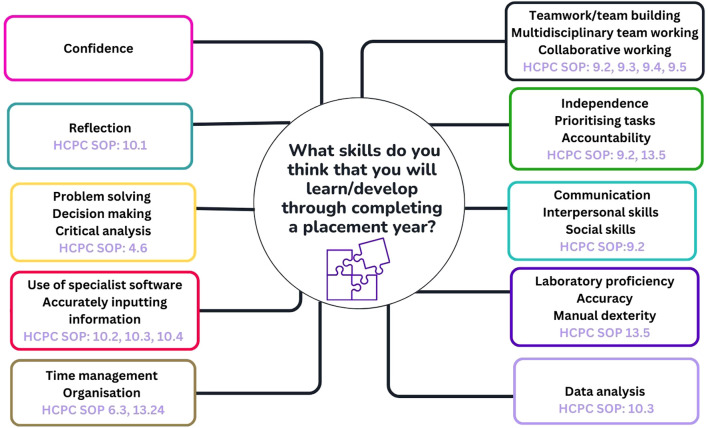
Transferable skills students recognise they will develop through completing a placement year. These have been mapped to HCPC Standards of Proficiency for Biomedical Scientists [6].

Students were asked to include examples of the transferable skills they believed they would gain upon placement completion (Figure 5). Specific comments included:

“Development of valuable transferable skills such as communication, teamwork, using initiative, and leadership. With COVID-19 and lockdown having an impact on the number of opportunities available to young people at an important stage of life, many students may not have the experience of part-time work or confidently speaking to different people, therefore will not have yet developed these transferable skills.”

“Completing a placement year in an NHS hospital laboratory offers invaluable hands-on experience in a clinical setting, allowing me to develop essential laboratory skills whilst working with real patient samples. Beyond career benefits, a placement year in an NHS laboratory would enhance problem-solving, adaptability and resilience in preparation for the fast-paced and high-pressure nature of diagnostic work.”

## Discussion

Previous work has longitudinally explored the impact of undergraduate work placements on graduate employment outcomes and found that whilst work placements are linked to improved job prospects, there are inequities in access. It underscores the importance of identifying and addressing barriers that prevent equitable participation in placements, and the need to understand barriers experienced by specific student cohorts and provide targeted support in accessing work placement opportunities.

Reports have revealed that completing a placement year is associated with significantly improved academic and employment outcomes. Students who undertook a placement were more likely to achieve a First-class degree and reported higher graduate prospects compared to those who did not [22]. This research sought to identify the barriers biomedical science students face when choosing to complete a placement year, with a view to developing strategies which promote inclusivity to overcome these barriers in the future. Through these strategies, it is hoped that more students can engage with the placement process, which in turn will benefit the future biomedical science workforce.

### Students Recognise the Value of Placement

Students clearly recognised the benefits of undertaking a placement year and identified a range of key transferable skills they expect to develop through clinical placement including teamworking, independence, communication skills, problem solving, data analysis, decision making and time management to name a few (Figure 5). These anticipated skills closely align with many of the HCPC Standards of Proficiency for Biomedical Scientists [6].

Previous studies have reported similar improvements in students’ transferable skills following a placement year. Students who have completed a placement year are more confident about their transferable skills [7, 9] and are more likely to secure graduate employment [12, 13, 23]. Students who have completed a placement year believe that they have improved oral and written communication and interpersonal skills, with many students stating that they feel more confident with expressing their opinion after placement [8]. In addition, as students progress through the placement selection process (including application and multiple interview stages), they develop key skills which will support them in obtaining graduate employment [12]. Other transferable skills that were identified by students following a placement year include improved organisational skills, team working skills, increased subject knowledge, a better understanding of ethical issues and development of higher academic skills [8] as well as development of critical thinking skills [23]. Student attendance at taught sessions is often higher following a placement year and they are more likely to engage with their lecturers and feel more confident to express their opinion amongst peers [11].

### Factors Influencing Placement Uptake

The socioeconomic background of West Midlands universities is diverse, with a high proportion of students enrolling from lower socioeconomic backgrounds [24, 25]. Furthermore, a large proportion of students identify from global majority backgrounds, which is reflected in the study participants where 73.8% were from diverse ethnic backgrounds. Studies have shown that global majority students on some courses are less likely to complete a placement year [22]. However, in this study participants from global majority backgrounds were not disadvantaged in obtaining a placement. This demonstrates the rigorous processes that have been implemented by West Midlands universities to promote inclusivity during the placement application stages. Global majority students often face challenges in securing placements due to a combination of limited access to professional networks, structural inequalities and unconscious bias [26, 27]. More broadly across the higher education setting, despite increasing efforts to widen participation, data consistently shows disparities in admission rates, progression, and work placement opportunities between global majority students and their white counterparts. According to a recent report, ethnic minority graduates from lower socio-economic backgrounds are 45% less likely to secure entry-level professional roles compared to their white peers [28].

An emerging theme identified through the quantitative and qualitative analysis is the lack of funding associated with placement opportunities. Financial constraints are a significant deterrent for students considering placements, particularly when these opportunities require relocation or incur additional living expenses (Figure 4). One study focusing on health students during the COVID-19 pandemic revealed that over half of the respondents reported financial concerns, with many citing the costs of travel, accommodation, and the inability to maintain paid employment during placements as substantial burdens [29]. Many students pursue part-time work alongside their studies which allows them to develop essential transferrable skills which are important to future employers. For students completing a placement year on an Applied Biomedical Science course, the daily workload, alongside the need to complete the Registration Training portfolio makes it challenging for students to continue with paid part-time employment. It is well recognised that unpaid placements provide a challenge for students who need to continue with part-time employment for financial reasons [8]. This financial pressure may prevent some students from pursuing these opportunities, resulting in unpaid placements becoming only accessible to students financially supported by their families. The universities within the West Midlands typically attract locally domicile students who have geographic restrictions further impacting their abilities to apply to placement opportunities outside of their local region.

The importance of the financial challenges associated with a placement year was a particularly strong theme amongst the Level 6 students who had experience of juggling their finances during a placement year. This challenge is further exacerbated for students that have childcare and caring responsibilities. This study found that a greater proportion of individuals with caring responsibilities opted not to apply for a placement, compared to those without caring responsibilities. Previously, students have highlighted the challenges associated with completing a full-time placement alongside caring responsibilities, particularly in health professions [19]. Students with caring responsibilities may find a full-time unpaid placement too demanding to participate. Despite the challenges of completing a placement alongside caring responsibilities, students with these responsibilities are seen as highly motivated with good time management skills [19].

Furthermore, only 33% of students who disclosed a disability indicated they would apply for a placement regardless of whether it was paid, compared to 42% of students who did not disclose a disability. It is well recognised that students with a disability are disadvantaged in the job market and when applying for placement opportunities [30]. Disclosure of a disability is often a source of anxiety for students, and this can provide a barrier for some individuals [31]. This study suggests that the absence of financial support may act as a deterrent for students with disabilities. These findings are in line with other studies which have also stressed the financial burden of placements [32–34]. Whilst disabilities were identified as an important barrier, the findings are based on self-reported data from a small and heterogeneous group of students and should therefore be interpreted with caution. The authors did not have ethical approval to link individual disability categories to institutional records, and undertaking such an analysis would likely constitute a separate, substantial project. It is also important to note that universities only hold disability data for students who self-declare; consequently, students who do not disclose a disability would not be captured in institutional records. Further work is needed to explore in greater detail the specific barriers faced by students with disabilities.

### Financial Challenges and the Impact of Modernising Scientific Careers

Prior to 2012, NHS funded courses, including biomedical science degrees leading to HCPC registration as a Biomedical Scientist, attracted a non-means tested HEFCE bursary to provide financial support for trainee Biomedical Scientist placements within the NHS. This bursary was withdrawn in 2012 which coincided with the introduction of PTP programmes as part of Modernising Scientific Careers (MSC). In the UK, several IBMS Accredited PTP degrees were introduced, however most have since been withdrawn as they struggled to meet service needs [32]. This is largely due to the structure of PTP clinical placements which are not easily accommodated by laboratories. The one-year NHS trainee Biomedical Scientist placements meets pathology needs and equips students with the necessary training required to become HCPC registered Biomedical Scientists post-graduation. Unfortunately, despite the decline of PTP degrees, as biomedical science programmes and pathology training places are not commissioned, they fail to attract any central funding for students or host laboratories. Recognising this, the 2023 IBMS Long Term Workforce Plan makes recommendations for the introduction of registration training grants for departments to train individuals completing their IBMS Registration Training Portfolio [32]. Despite the IBMS recognising that central funds are not available to students, there are currently no recommendations related to providing funding opportunities for trainee Biomedical Scientists.

### Increasing Competition for Limited Placement Opportunities

The challenge of securing appropriate placement opportunities is further intensified by competitive labour markets and the limited availability of roles. This is especially evident in the case of trainee Biomedical Scientist positions within pathology laboratories, where demand significantly exceeds supply. For instance, whilst the West Midlands Applied Biomedical Science Placement Programme successfully facilitates the creation of approximately 40 trainee Biomedical Scientist positions annually, these opportunities are highly oversubscribed, currently with over 200 students from five HEIs competing for placement. For the first ten years, these placement opportunities were exclusively available to three HEIs (Aston, Coventry and Wolverhampton universities) who worked in collaboration with local training officers to design and deliver tasks to fulfil the requirements of the IBMS Registration Training portfolio. Post COVID-19, Keele and Staffordshire universities joined the West Midlands Regional Training Officers (WMTO) group thus increasing competition for the limited pool of placement opportunities. With HEIs increasingly recognising the benefit of an IBMS accredited degree, the number of HEIs participating in this collaboration is forecast to increase further by 2026. Consequently, a substantial proportion of students miss placement years entirely or are compelled to seek alternative placements that may not align as closely with their academic and professional aspirations.

It is important to consider how awarding gaps and inequities in academic achievement may influence access to trainee Biomedical Scientist placements in West Midlands pathology laboratories. Placement allocation is highly competitive, and academic performance plays a role in shortlisting candidates. Each university has defined pre-placement criteria, including a minimum pass rate requirement for Level 5, attendance at placement workshops, and successful completion of all Level 5 assessments. Differential attainment across student groups may therefore contribute to unequal opportunities for securing placements. Furthermore, the barriers identified in this study are unlikely to act in isolation. Students may experience multiple, overlapping factors, such as financial pressures, caring responsibilities, disability, or geographical constraints, which interact and compound their impact. Recognising this intersectionality is essential for understanding cumulative barriers to placement participation and for informing more equitable approaches to allocation and support within the region.

In the West Midlands area, there has been a consistent increase in the number of students aspiring to undertake Biomedical Scientist placements. However, this growing demand has not been met with a proportional expansion in available pathology laboratory placements. A contributing factor to this disparity is the limited capacity of current training officers, coupled with a shortage of Biomedical Scientists able to take up additional supervisory roles on top of their existing responsibilities. The IBMS Long Term Workforce Plan highlights these challenges, emphasising the need to expand training positions and introduce registration training grants to support departments training individuals completing their IBMS Registration Training Portfolio [35]. Furthermore, the IBMS underscores the importance of strategic workforce planning to ensure adequate staffing and expertise within community-based diagnostic services, advocating for investment in pathology networks to enhance collaboration and optimise capacity across NHS trusts. Addressing these issues is crucial to align educational aspirations with practical training opportunities, thereby ensuring a sustainable pipeline of qualified Biomedical Scientists [36].

### Recommendations

This study has demonstrated that financial concerns are a significant barrier for students when considering whether to apply for a placement year. This barrier is greater for those students with caring responsibilities and those that declare a disability. Following this study, it is strongly recommended that the NHS Business Services Authority (BSA) review the NHS Learning Support Fund (LSF) grant and include Biomedical Scientists into the pool of professionals that are eligible for funding to reduce the inequity described. Currently, the NHS LSF supports students on a range of health-related courses, including nursing, midwifery, physiotherapy and other allied health professions, by providing a non-means tested training grant of £5,000 per student per year [37]. Although the placement year is not a compulsory component for biomedical science courses, this financial incentive would be welcomed by students. Biomedical Scientists are pivotal to providing patient-centred care; however, they are the only HCPC registered profession that complete degree courses without access to this funding. This demonstrates a stark disparity in the education and training of this essential workforce.

In addition, those students who are eligible for the training grant are also eligible to apply for Travel and Dual Accommodation Expenses (TDAE) [38]. This travel grant allows students to fund temporary accommodation when it is not practical for them to travel from their term-time address to placement daily. Students are also eligible to claim daily travel costs to placement if they exceed normal travel costs for getting to university. For students studying Biomedical Science, this would help to alleviate the financial pressures of the placement year and to provide support with travel costs, which 95.3% of participants recognised as an important factor when deciding whether to apply for a placement year.

Financial pressures, travel costs and the need to balance part time work have been identified as potential barriers in other courses which contain a placement [31]. Allowing Biomedical Science students completing an NHS placement year to access both NHS LSF grant and TDAE funding would significantly reduce the barriers that students currently face. This would help to reduce inequities within the profession, as currently only those students who are supported financially are able to pursue a placement year. This funding may also allow those students with disabilities and caring responsibilities equitable access to a placement year.

Only approximately 40 trainee Biomedical Scientist placements are offered within the West Midlands region of England annually and the number of IBMS accredited degree programmes is expanding. The introduction of funding for laboratories that support trainee Biomedical Scientists to complete the IBMS Registration Training portfolio will be a positive step for the future workforce [35]. It is hoped that this funding will encourage laboratories to consider supporting a greater number of candidates to complete the Registration Training portfolio to fulfil the workforce requirements of the region and ensure that students on Applied Biomedical Science courses are able to enter the profession. Furthermore, NHS Trusts should actively collaborate and engage in strategic succession planning to ensure that sufficient placement opportunities are made available each year in response to evolving workforce requirements. Additionally, the capacity to deliver high-quality training should be formally incorporated into establishment budgets to support sustainable workforce development.

This study has also highlighted that students with disabilities and caring responsibilities find accessing placements more challenging. Although financial pressures may be an important part of this, further research is required to identify why individuals in these groups may choose not to apply for a placement year. Once these factors have been identified, strategies can be implemented to provide equitable access for all. The full-time nature of the placement year and the intensity of balancing work and home life during this period may serve as a barrier for those with disabilities or caring responsibilities. It is essential for universities to consider how to best support students from certain under-represented groups to access placement due to the associated benefits for graduate employability [22].

### Limitations of the Study

Although the study was successful in seeking the views of students studying BSc Biomedical Science at undergraduate level, there are several limitations which must be acknowledged. Firstly, the sample size is relatively small with 107 participants across Levels 5 and 6. Whilst quantitative research is typically focused on sample size, for qualitative or mixed methods research, the focus is on obtaining a representative sample of participants [39]. One of the strengths of the study is that students from four universities participated, meaning that the findings are not just applicable to a single institution. The importance of transferability in qualitative and mixed methods research, where the findings from specific research participants are applied to those not studied directly, is an important consideration for guiding practice and policy [40]. Therefore, this collaborative approach increases transferability and the number of potential participants in a study. However, across the institutions there were numerous students who did not participate, and the response rate was disappointing (16.4% at Level 5% and 15.7% at Level 6). Survey based research projects can be challenging to recruit sufficiently large numbers of participants, with 44% considered the average response rate in the education field [41]. Although this response rate was moderate, the observed patterns applied across all HEIs suggesting that the responses capture relevant feedback from across the student cohort.

Although this study focuses on one region, the issues identified may not be confined to the West Midlands. Through the IBMS Higher Education Learning and Practice (HELP) support sessions delivered by the authors to multiple HEIs in the UK, similar concerns regarding placement access, financial pressures, and differential opportunities have been consistently raised, suggesting that many of these challenges may be more widespread. It is also important to note that the lack of dedicated NHS funding for placement years affects all Biomedical Science students nationally, not only those within this regional sample. Further work is underway to extend this research to additional regions, including institutions in the north of England, to explore whether the patterns observed here align with the broader national landscape.

It was challenging to recruit participants to this study, which is possibly related to the timing of the survey distribution or the lack of a financial incentive to participate. Data collection was unfortunately delayed whilst awaiting ethical approval from each of the participating universities. As a result, data collection took place during February 2025 and feedback from potential Level 6 participants suggested they were too busy completing their dissertations and managing assignment submission deadlines. This may have reduced the number of responses that were received, particularly at Level 6. Where possible, students were given time during a taught session to complete the questionnaire to reduce the pressure of conflicting deadlines as data collection is more effective when conflicting pressures are decreased [42]. This study was carried out without internal or external funding, meaning students were not offered any incentive to participate. This may have reduced the number of responses that were received as both financial and non-financial incentives are known to increase participation [43, 44].

In addition, the ethical approvals associated with the study allowed students to omit responses to any questions they did not wish to answer. Whilst this was considered ethically important to allow students free choice when answering a question, it resulted in some incomplete responses. In particular, the free-text responses were left blank in some cases. When participants omit responses, it can be through a deliberate choice or through unintentionally missing out a question. These non-responses are important to capture and represent a valid participant response. Although JISC Online Surveys allows all questions to be mandatory, forcing a participant to give a response may lead to biased or inaccurate data [42]. Questionnaire research methodology is negatively impacted by Insufficient Effort Responding (IEF), whereby research participants provide reduced effort when completing a questionnaire which reduces the quality of the study data [45]. However, in a mixed-methods study, these free-text responses are an important data source to provide rich, high-quality data for thematic analysis [46].

Finally, a significant proportion of the participants (49%) identified as having completed a placement year or intended to complete a placement year. Although it was made clear that the study was open to all students regardless of placement status, a greater proportion of participants had completed a placement than typically observed. This may be because participants are more likely to participate in a study that they have a vested interest in [47, 48]. Those students who have personal experience of a placement were potentially more likely to participate in the study due to the perception that they have a relevant opinion to contribute [47]. However, as this study was interested in the decision making involved in pursuing a placement year, the opinions of individuals who had not completed a placement were also sought. It was unfortunate that there were fewer responses from those who had not completed a placement year, as this was the population with arguably the most relevant opinion to the study. As a result, the findings of the study should be interpreted with caution due to the potential bias of participants who had completed a placement year.

### Conclusion

In conclusion, this study identifies key barriers to placement uptake amongst undergraduate biomedical science students including geographical restrictions, caring responsibilities, financial pressures, placement length and disabilities. Many of these exacerbate financial strains, discouraging students from applying to trainee Biomedical Scientist positions. Whilst there is a growing need to increase the Biomedical Scientist workforce, the strategies to increase training opportunities and ensure becoming a Biomedical Scientist remains attractive are not as well established. The IBMS recognise the importance of providing a framework for growth, however the associated funds are not as clearly defined. Therefore, equitable, centralised funding is key to support all healthcare students, including Biomedical Scientists. Through access to funding, marginalised populations of students will have the same opportunities as their peers, therefore reducing academic barriers and producing more employable graduates to meet pathology workforce demands.

### Concluding Statement

This work represents an advance in biomedical science because Biomedical Science students face barriers and have less equitable placement opportunities, due to lack of centralised funding.

## Summary Table

### What Is Known About This Subject:

Placements allow completion of the IBMS Registration Training portfolio leading to HCPC registration post-graduation.

The West Midlands Biomedical Science consortium creates approximately forty placement opportunities annually.

There are fewer placement opportunities available compared to students wishing to pursue a biomedical science placement.

### What This Paper Adds:

Availability of local placements and funding is a significant barrier to placement uptake for biomedical science students.

IBMS support is vital for pathology laboratories to host additional biomedical science placements to meet workforce demands.

NHS BSA should review the Learning Support Fund grant to include biomedical science courses for funding to reduce inequity.

## Data Availability

The original contributions presented in the study are included in the article/supplementary material, further inquiries can be directed to the corresponding author.
